# Circulating anti-filamin C autoantibody as a potential serum biomarker for low-grade gliomas

**DOI:** 10.1186/1471-2407-14-452

**Published:** 2014-06-18

**Authors:** Masayo Adachi-Hayama, Akihiko Adachi, Natsuki Shinozaki, Tomoo Matsutani, Takaki Hiwasa, Masaki Takiguchi, Naokatsu Saeki, Yasuo Iwadate

**Affiliations:** 1 Department of Neurological Surgery, Chiba University, Graduate School of Medicine, 1-8-1, Inohana, Chuo-ku, Chiba 260-8670, Japan; 2 Department of Biochemistry and Genetics, Chiba University, Graduate School of Medicine, Chiba, Japan; 3 Division of Neurosurgery, Narita Red-Cross Hospital, Iida-cho, Narita 286-8523, Japan

**Keywords:** Glioma, Filamin C, FLNC, Biomarker, Early diagnosis

## Abstract

**Background:**

Glioma is the most common primary malignant central nervous system tumor in adult, and is usually not curable due to its invasive nature. Establishment of serum biomarkers for glioma would be beneficial both for early diagnosis and adequate therapeutic intervention. Filamins are an actin cross-linker and filamin C (FLNC), normally restricted in muscle tissues, offers many signaling molecules an essential communication fields. Recently, filamins have been considered important for tumorigenesis in cancers.

**Methods:**

We searched for novel glioma-associated antigens by serological identification of antigens utilizing recombinant cDNA expression cloning (SEREX), and found FLNC as a candidate protein. Tissue expressions of FLNC (both in normal and tumor tissues) were examined by immunohistochemistry and quantitative RT-PCR analyses. Serum anti-FLNC autoantibody level was measured by ELISA in normal volunteers and in the patients with various grade gliomas.

**Results:**

FLNC was expressed in glioma tissues and its level got higher as tumor grade advanced. Anti-FLNC autoantibody was also detected in the serum of glioma patients, but its levels were inversely correlated with the tissue expression. Serum anti-FLNC autoantibody level was significantly higher in low-grade glioma patients than in high-grade glioma patients or in normal volunteers, which was confirmed in an independent validation set of patients’ sera. The autoantibody levels in the patients with meningioma or cerebral infarction were at the same level of normal volunteers, and they were significantly lower than that of low-grade gliomas. Total IgG and anti-glutatione S-transferase (GST) antibody level were not altered among the patient groups, which suggest that the autoantibody response was specific for FLNC.

**Conclusions:**

The present results suggest that serum anti-FLNC autoantibody can be a potential serum biomarker for early diagnosis of low-grade gliomas while it needs a large-scale clinical study.

## Background

Glioma is the most common type of primary brain tumor in adults. Currently, tumor grading by histological analysis of surgically-resected specimens is the most reliable predictor of glioma prognosis. They are classified into four grades; low-grades including WHO Grade I (localized gliomas) and WHO Grade II (diffuse gliomas), and high-grades including WHO Grades III (anaplastic gliomas) and WHO Grade IV (glioblastoma). Despite the recent advances in glioma diagnosis and therapy, two-year survival for the grade IV glioblastoma is less than 30%. Even among patients with low-grade gliomas that usually confer a relatively good prognosis, treatment is almost never curative under the current diagnostic system [1].

Identification of specific glioma antigens is long awaited for the clinical management such as early diagnosis, more objective diagnosis, monitoring treatment response, and for novel therapeutic targets for glioma [2-5]. In the previous study utilizing proteomics, we found several proteins that are overexpressed in high-grade gliomas and some were potentially applicable to serum biomarkers by ability of secretion [6]. Serum levels of these candidate proteins were shown to correlate significantly with tumor grade, invasive nature of the tumor and patient survival periods [7,8]. However, detection of low-grade tumors is difficult by utilizing the protein amount-based diagnostic system because their serum protein levels may not be sufficiently altered to be detectable by current proteomic technologies. One approach for overcoming this difficulty and thereby enable early detection of slight changes in protein amount, protein structure or protein localization would be utilization of antigen-antibody interactions [9,10]. Detection of the host immune reactions which can respond to slight changes in the precursor cells that have started to transform into neoplastic cells would be a breakthrough to enable early diagnosis of cancers including glioma. To verify this concept, we utilized immunoscreening of cDNA libraries prepared from glioblastoma cells with IgG in the sera from glioma patients.

We found filamin C (FLNC), which is normally restricted in muscle tissues but abundantly exists in fetal central nervous system [11,12], as a candidate protein for glioma antigen. Filamins are an actin cross-linker, and serve as scaffolds for many binding partners including channels, receptors, intracellular signaling molecules, and transcription factors [13,14]. Because of these extensive fields of associating proteins, mutations in filamin genes result in a wide range of cell and tissue anomalies. Especially they have a decisive role in cellular motility and migration [11-14]. Furthermore, Filamin genes mutations are common in human breast and colon cancers [15]. Many recent studies have suggested filamin A as an important factor for tumor malignancy and invasiveness in various human cancers including primary brain tumors [16-20]. In addition, filamin A interacts with BRCA1/2 or other DNA repair-related proteins to affect the DNA repair process resulting in resistance to radiation and chemotherapy [21,22]. In this paper, we examined tissue expressions of FLNC (both in normal and tumor tissues), and investigated the serum levels of anti-FLNC autoantibody in glioma patients.

## Methods

### Sera and tissue specimens

We analyzed 131 glioma patients’ sera (low-grade, 72; high-grade, 59) along with 77 sera from healthy volunteers, 19 sera from patients with meningioma, and 24 patients with cerebral infarction at chronic stage. These were newly-diagnosed patients, and had no other cancer or diseases at the time of sample collection. They had serum drawn at the time of initial diagnosis. The patient demographics and clinical profiles are presented in Table 1. Forty-eight glioma tissues surgically-resected from newly diagnosed glioma patients (low-grade, 22; high-grade, 26) and 10 healthy brain tissues were analyzed for the tissue expression of FLNC. The normal brain tissues were obtained from the patients undergoing resection of extra-axial brain tumors or epilepsy surgery. Sixty-five serum samples from glioma patients and 38 samples from healthy volunteers were used to develop a diagnostic model (training set) that was validated in an independent, blinded validation set using the serum samples from 66 glioma patients and 39 healthy volunteers. The protocol of this study was approved by the Institutional Review Board of Chiba University, and written informed consent was obtained from the patients or their guardians. Total RNAs of the lung, liver, spleen, testis and muscle were commercially obtained (Zyagen Laboratories, San Diego, CA). Total RNAs of lung, liver, spleen, testis and muscle were commercially obtained (Zyagen Laboratories, San Diego, CA).

**Table 1:** Characteristics of the training set and validation set

	**Training set**		**Validation set**	
**Demographic**	**Glioma (n=65)**	**Control (n=38)**	**Glioma (n=66)**	**Control (n=39)**
Age				
Mean	46.6	47.6	49.2	51.2
Range	12-74	34-68	24-78	22-77
Sex				
Male	40	21	37	21
Female	25	17	29	18
WHO grade				
I	7		8	
II	28		29	
III	9		9	
IV	21		20	

### Serological analysis of recombinant cDNA expression libraries (SEREX)

Total RNA was prepared from the U87MG glioblastoma cell line by the acid guanidium thiocyanate-phenol-chloroform method, and purified to poly(A) + RNA using the Oligotex-dT30 (Super) mRNA Purification Kit (Takara Biochemicals, Kyoto, Japan). cDNA was ligated into the EcoRI-XhoI site of the λZAP II phage. The original library size was 1 × 10^6^. *Escherichia coli* XL1-Blue MRF’ was infected with the λZAP II phages which contained the U87MG cDNA library, and the expression of cDNA was induced by blotting on nitrocellulose membranes which had been pretreated for 30 min with 10 mM IPTG (Wako Pure Chemicals, Osaka, Japan). After washing and blocking, the membranes were exposed in 1:2000-diluted sera from 18 glioma patients. Then, the membranes were treated with 1:5000-diluted alkaline phosphatase-conjugated F(ab)’ fragment-specific goat antihuman IgG. Positive reactions were detected by incubation in a color development solution containing 0.3 mg/mL of nitroblue tetrazolium chloride and 0.15 mg/mL of 5-bromo-4-chloro-3-indolyl-phosphate. Positive clones were re-cloned twice to obtain monoclonality and retested for the serum reactivity.

### Sequence analysis of identified antigens

Monoclonalized phage cDNA clones were converted to pBluescript phagemids by in vivo excisions with ExAssist helper phage (Stratagene, La Jolla, CA). Plasmid DNA was obtained from E. coli SOLR strain transformed by the phagemid. The cDNA inserts were sequenced by the dideoxy chain termination method using the DNA sequencing kit BigDye Terminator (Applied Biosystems, Foster City, CA). Sequences were analyzed for homology with public databases of known genes and proteins using BLAST on the National Center for Biotechnology Information’s website (http://www.ncbi.nlm.nih.gov/gene or protein).

### Purification of recombinant FLNC protein

The cDNA insert of FLNC incorporated in pBlueScript was cleaved by EcoRI and XhoI, and then recombined in pGEX-4 T-3. E. coli JM109 cells containing either pGEX-4 T-3- FLNC or control pGEX-4 T-3 were cultured in 200 mL of Luria broth and treated with 1 mM IPTG for 2.5 hrs. The cell lysate was centrifuged and GST-FLNC in the supernatant was directly purified with glutathione- Sepharose (Amersham Biosciences, Piscataway, NJ). The purified proteins were concentrated using Apollo centrifugal concentrators (Orbital Biosciences, Topsfield, MA).

### ELISA for anti-FLNC autoantibody

Fifty μl of antigen (GST or GST-tagged recombinant FLNC) was added to each well, and incubated at 4°C overnight. The plate was washed and blocked with 10% fetal calf serum in PBS (PBS-FCS). Fifty μl of sera diluted at 1:100 in 10% PBS-FCS was added to the wells and then they were incubated. The bound IgG antibodies were detected by incubating with horseradish peroxidase-conjugated antihuman IgG antibody (Jackson Immuno Research Laboratories, West Grove, PA), followed by the addition of 100 μl of a peroxidase substrate (*o*-phenylenediamine, 0.4 mg/ml) in a citrate-phosphate buffer. Absorbance at 490 nm was determined using a microplate reader (Emax; Molecular Devices, Sunnyvale, CA).

### Sandwich ELISA for Serum FLNC measurement

ELISA 96-well plates were coated with 20 μg/ml antihuman FLNC antibody, and were filled overnight with 50 μl of patients’ sera diluted 1:100. The plates were developed with o-phenylene-diamine (Sigma-Aldrich, St Louis, MO) and were read at an absorbance of 490 nm.

### Total IgG measurement

Serum total IgG was measured in the same samples as the anti-FLNC autoantibody measurements according to the manufacturer’s instructions using a human IgG ELISA quantitative kit (Bethyl, Montgomery, TX).

### Extraction of mRNA and preparation of cDNA

The mRNAs were extracted from the tumors and normal brain tissues using the QIAzol Lysis Reagent and RNeasy^®^ Lipid Tissue Mini Kit (QIAGEN, Tokyo, Japan), followed by DNase treatment. One μg of each mRNA was reversely transcribed using the oligo dT primer (Takara Biochemicals, Inc., Tokyo, Japan) and Super Script II (Invitrogen, CA).

### Real-time RT-PCR

The real-time quantitative RT-PCR with SYBR-green was performed using the Light Cycler (Roche Diagnostics, Meylan, France). The amplification was performed using 5’-GGACATGAGTGGCCGGTACAC-3’ as the forward primer and 5’-ACTGTGACGAGGCACTTGCTG-3’ as the reverse primer. A series of cDNA dilutions, 1/1, 1/10, 1/100, and 1/1000, were used in each run separately. Standard curves were obtained by doing serial dilutions of the same sample in each run. Then, 1 mM of each primer and 3 mM of MgCl_2_ in the total volume of 20 μl were used. The real-time RT-PCR cycle started with the initial denaturation at 95°C for 10 min, followed by 45 cycles of denaturation at 95°C for 10s, annealing at 61°C for 10s and then elongation at 72°C for 10s. As an internal quantitative control of the gene expression, the glyceraldehydes-3-phosphate dehydrogenase *(GAPDH)* gene expression was used. The ratios of *filamin C* and *GAPDH* gene expressions represented the normalized relative levels of *filamin C* expressions.

### Immunohistochemistry

IHC staining was performed on 4 μm paraffin-embedded sections. Antigenicity was recovered by the microwave method. Endogenic peroxidase was inactivated with 0.3% H_2_O_2_ methanol. After antigen blocking, the sections were incubated overnight with mouse monoclonal primary antibody against FLNC (Lab Vision, Fremont, CA). The sections were then incubated with mouse biotinylated secondary antibody followed by the ABC complex reaction. Finally, the reaction was visualized using DAB and counterstained with hematoxylin. To quantitate FLNC protein expression, the mean percentage of positive tumor cells was determined in at least 5 random fields at x400 magnification in each section.

### Statistical analysis

Results of ELISA were statistically analyzed by unpaired *t*-test. Receiver–operating characteristics (ROC) curve analysis was used to determine the optimal cutoff values for differential diagnosis of low-grade gliomas and healthy volunteers. The survival rates were estimated using Kaplan-Meier method, and they were compared with the log-rank test. The correlation between the filamin C mRNA expression levels and serum anti-FLNC autoantibody concentrations was analyzed using the non-parametric Spearman’s rank test. The statistical analyses were performed using Stat-View software and SAS software (SAS Institute Inc., Cary, NC).

## Results

Screening of the patients’ sera by serological analysis of recombinant cDNA expression libraries (SEREX) resulted in identification of filamin C (FLNC) as one of the candidate glioma antigens (Additional file 1: Table S1). The list included many signal-transduction molecules and transcription factors, which was also confirmed in a previous proteomic study.

**Figure 1:**
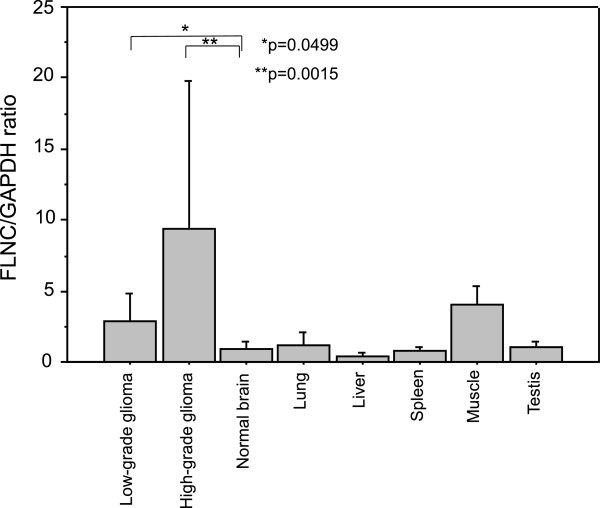
**Tissue expression of *filamin C *mRNA was measured by quantitative real-time RT-PCR analysis in normal brain tissues, low-grade gliomas, and high-grade gliomas, and also in lung, liver spleen, testis and muscle which were commercially obtained.** The *filamin C* mRNA expression was significantly up-regulated in high-grade gliomas compared with normal brain tissues. It was moderately upregulated in low-grade gliomas and normal muscle tissues. The mean values of duplicate experiments for each sample are presented.

**Figure 2:**
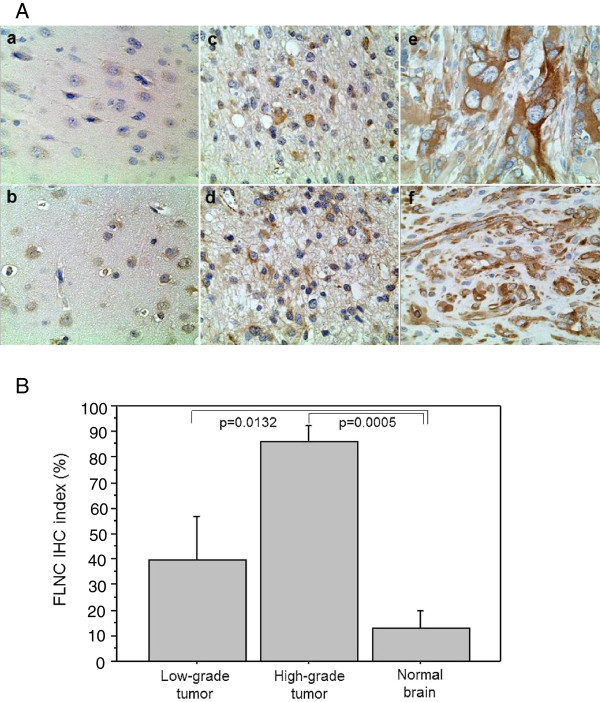
**Immunohistochemistry (IHC) for FLNC among normal brain, low-grade glioma and high-grade glioma. (A)** FLNC protein expression level was higher in high-grade glioma **(e, f)** than in low-grade glioma **(c, d)** which expressed significantly higher level of FLNC than normal brain tissues **(a, b)**. FLNC expression is only observed around the nucleus of glia cells in normal brains, whereas it spreads into the whole cytoplasm and the fibrous cellular processes of the glioma cells (magnification × 400). **(B)** Quantitative analysis of FLNC protein expressions in IHC shows that the mean percentage of positive tumor cells gets higher as the tumor grade advances. (normal brain vs low-grade glioma; p = 0.0132, low-grade glioma vs high-grade glioma; p = 0.0005).

We first examined whether the expression of *filamin C* mRNA is elevated in the glioma tissues (Figure 1). Quantitative reverse transcription–PCR (qRT-PCR) analysis of various glioma tissues and normal brain tissues confirmed that *filamin C* mRNA expression was significantly up-regulated in low-grade gliomas compared with normal brain tissues. High-grade gliomas expressed higher level of *filamin C* mRNA than low-grade gliomas. Other normal tissues including lung, liver, spleen, and testis contained the same levels of *filamin C* mRNA as normal brain tissues. In contrast, muscle tissues had a higher level than the other normal tissues and the same level as low-grade glomas (Figure 1).We then analyzed protein expression levels and distributions in paraffin-embedded clinical specimens utilizing semiquantitative immunohistochemical analysis (Figure 2). FLNC protein expression level was higher in high-grade glioma than in low-grade gliomas which expressed significantly higher level of FLNC than normal brain tissues (Figure 2-a, 2-b). FLNC protein expression was only observable around the nucleus of glial cells in normal brain tissue, but it spread into the whole cytoplasm and the fibrous cellular processes in the low-grade glioma cells (Figure 2-c).

**Figure 3:**
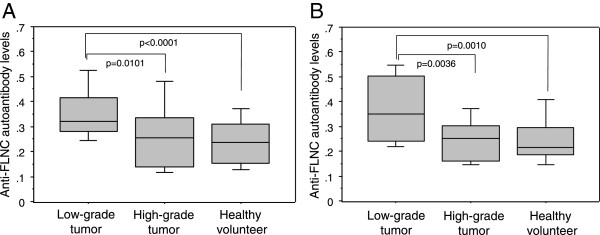
**ELISA of anti-FLNC autoantibody levels in the sera from glioma patients and normal volunteers. (A)** For a training set, anti-FLNC autoantibody concentration in the sera from low-grade gliomas was significantly higher than those from high-grade gliomas (p = 0.0101) or normal volunteers (p < 0.0001). **(B)** The ELISA result obtained in the training set was confirmed in an independent validation set (low-grade vs. high-grade: p = 0.0036, low-grade vs. healthy: p = 0.0010).

We measured anti-FLNC auto-antibody concentrations by ELISA in a training set consisting of 65 glioma patients (low-grade, 35; high-grade, 30) and 38 healthy volunteers focusing on FLNC (Table 1). The results showed the serum anti-FLNC autoantibody level was significantly higher in low-grade gliomas than in high-grade gliomas or healthy volunteers (low-grade vs. high-grade: p = 0.0101, low-grade vs. healthy: p < 0.0001) (Figure 3-a). We then analyzed the antibody level in a prospectively-collected validation set consisting of 66 glioma patients (low-grade, 37; high-grade, 29) and 39 healthy volunteers (Table 1). The significantly increased autoantibody level was confirmed in the patients with low-grade gliomas as compared to those in high-grade tumors or healthy volunteers (low-grade vs. high-grade: p = 0.0036, low-grade vs. healthy: p = 0.0010) (Figure 3-b). Although the categorization of grade I and grade II gliomas into low-grade gliomas is generally used in the clinical setting, these two are different diseases biologically and clinically. So, the anti-FLNC autoantibody levels were examined for each grade independently, which showed that those of grade I and grade II, and also of grade III and grade IV, were at the same levels (Table 2). To know whether the increase in anti-FLNC auto-antibody levels is specific to low-grade gliomas, the patients with other brain lesions whose MRI manifests a hypointesity on T1-weighted image and hyperintensity on T2-weighted image were examined. The anti-FLNC autoantibody levels for meningioma and cerebral infarction were at the same level of healthy volunteers (p = 0.2281, and p = 0.5581, respectively) (Table 2). The ROC curve analysis showed that a cutoff value of 0.31 provided the best sensitivity and specificity for the differential diagnosis between patients with low-grade gliomas and healthy volunteers. By using this cutoff value, the anti-FLNC autoantibody biomarker system correctly classified 52 (sensitivity; 72.2%) of 72 low-grade glioma patients and 62 (specificity; 80.5%) of 77 healthy volunteers (Table 3). Although the diagnostic ability was not sufficient for clinical application, the autoantibody-based tumor markers detected in the patients’ sera may contribute to the construction of early diagnosis system for low-grade gliomas.

**Table 2:** Sensitivity and specificity of anti-FLNC antibody

	**n**	**Mean value**	**Standard deviation**	**p-value for healthy volunteers**
Healthy volunteers	77	0.242	0.115	__
Low-grade gliomas				
Grade I	15	0.359	0.112	0.0021
Grade II	57	0.373	0.152	<0.0001
High-grade gliomas				
Grade III	18	0.254	0.159	0.9748
Grade IV	41	0.268	0.101	0.7503
Meningioma	19	0.273	0.081	0.2281
Cerebral infraction	24	0.273	0.082	0.5531

**Table 3:** Sensitivity and specificity of anti-FLNC antibody

	**n**	**Positive**	**Negative**
		**(absorbance ≥0.31)**	**(absorbance<0.31)**
Low-grade gliomas	72	52	20
Healthy volunteers	77	15	62

**Figure 4:**
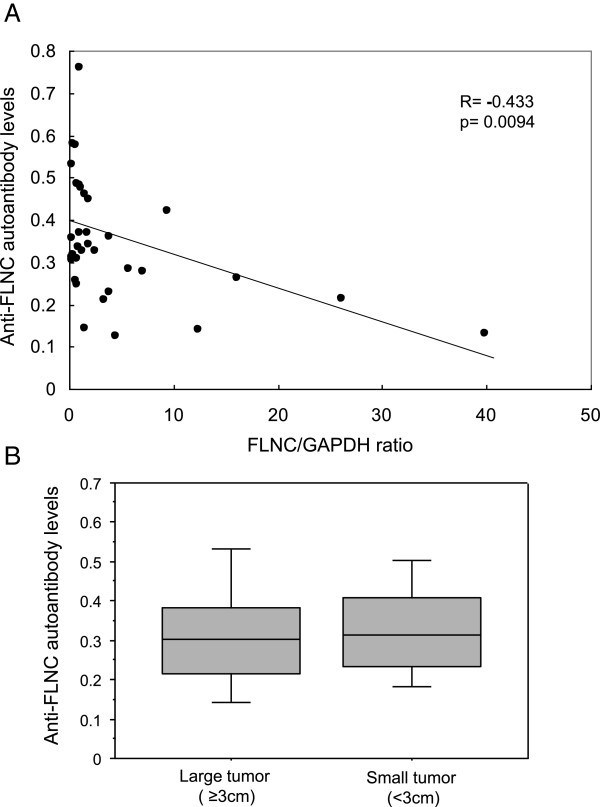
**Serum anti-FLNC autoantibody levels and glioma progression. (A)***Filamin C* mRNA expression level inversely correlated with the serum anti-FLNC autoantibody concentrations (p = 0.0094, R = -0.433). **(B)** Serum anti-FLNC autoantibody levels were not difference between large tumors (size ≥3 cm) and small tumors (size <3 cm).

**Figure 5:**
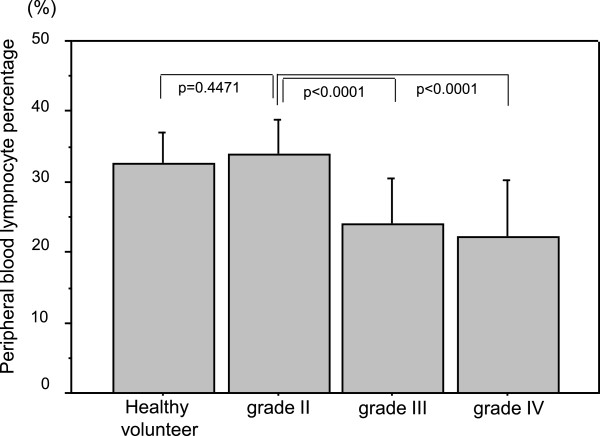
The mean lymphocyte ratio in the peripheral blood of grade II glioma patients was equivalent as healthy volunteers, but those in grade III and grade IV glioma patients were significantly decreased compared with healthy volunteers (both p < 0.0001).

**Figure 6:**
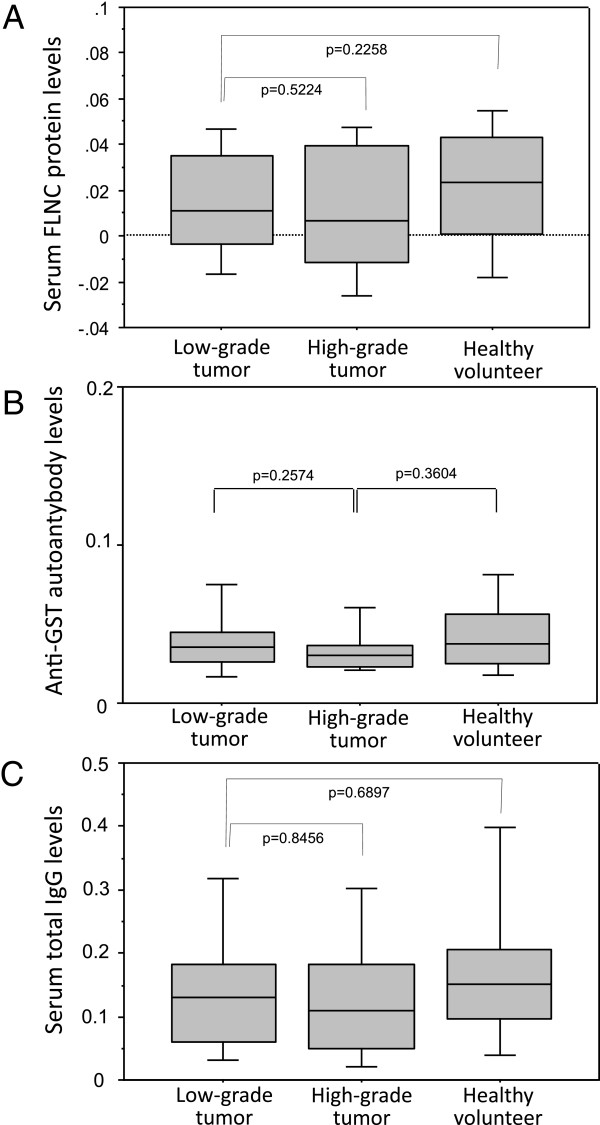
**Non-specific factors affecting serum autoantibody levels. (A)** Serum FLNC protein concentration levels were measured utilizing the sandwich ELISA to be proved equivalent among low-grade gliomas, high-grade gliomas and normal volunteers. **(B)** Serum total IgG and **(C)** ant-GST autoantibody levels were not different among low-grade gliom patients, high-grade glioma patients and normal volunteers.

**Figure 7:**
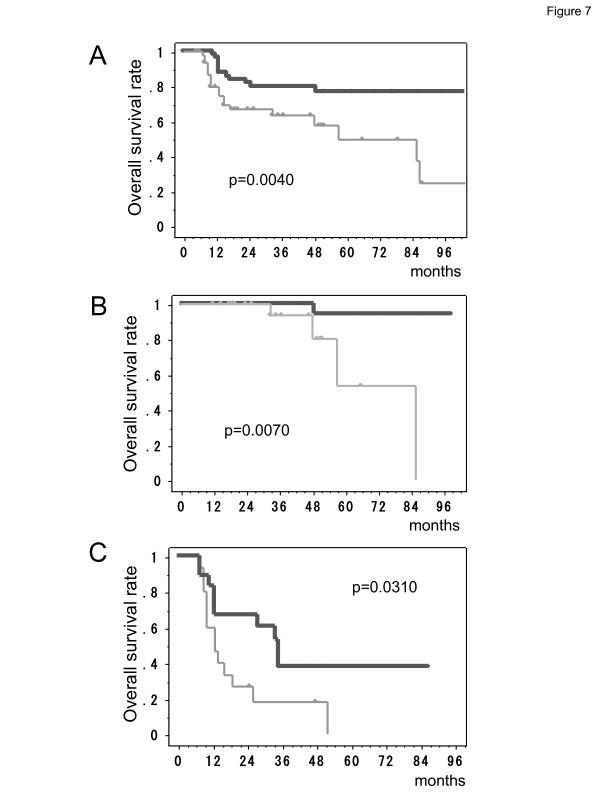
**Correlations of serum anti-FLNC autoantibody concentration measured by ELISA and survival of glioma patients.** Kaplan-Meier survival curves of glioma patients show that a group having higher values of serum anti-FLNC autoantibody (≥0.31) survived significantly longer than the other group with lower values **(A)** in all glioma patients (p = 0.0040), **(B)** in low-grade glioma patients (p = 0.0070), **(C)** and in high-grade glioma patients (p = 0.0310).

The *filamin C* mRNA expression level inversely correlated with the serum anti-FLNC autoantibody concentrations (p = 0.0094) (Figure 4-a). The serum anti-FLNC autoantibody levels showed no difference between large tumors (size ≥3 cm) and small tumors (size <3 cm) (Figure 4-b). These data collectively suggest that circulating anti-FLNC autoantibody is induced at the early stage of glioma progression, and the serum level decreases with tumor progression.There was a discrepancy between the decreased level of anti-FLNC autoantibody and the increased tissue expression of FLNC in the patients with high-grade gliomas. To investigate the mechanisms for the decreased level of anti-FLNC autoantibodies in high-grade glioma patients in spite of the high tissue expression, we first measured peripheral blood lymphocyte amount, which showed that the mean lymphocyte ratio in the peripheral blood of grade II glioma patients was equivalent level as healthy volunteers, but those in grade III and grade IV glioma patients were significantly decreased compared with healthy volunteers (both p < 0.0001) (Figure 5). The lymphocytopenia would contribute to the decreased anti-FLNC autoantibody level in high-grade glioma patients. Then, serum FLNC protein concentration levels were measured utilizing the sandwich ELISA to be proved equivalent among low-grade gliomas, high-grade gliomas and normal volunteers (Figure 6-a). Serum levels of total IgG and anti-GST autoantibody were also not different among the patient groups and normal volunteers (Figure 6-b and 6-c), which indicated that general B cell function was maintained and that the decreased level of anti-FLNC autoantibody production was not a non-specific reaction but was specific for FLNC.All the glioma patients included in this study were also subjected to survival analysis. Figure 7-a shows Kaplan-Meier overall survival curves for the two patients groups divided by the anti-FLNC antibody level of 0.31 in the ELISA. The group presenting higher anti-FLNC autoantibody levels achieved significantly longer survival periods (p < 0.01). When the survival analysis was focused on low-grade gliomas, the patients with higher anti-FLNC autontibody level (≥0.31) lived significantly longer than those with lower levels (Figure 7-b). Likewise in high-grade gliomas, the patients with higher antibody level had significantly longer survival periods (Figure 7-c). Serum anti-FLNC autoantibody is a useful predictor for longer survival periods in patients with both low-grade gliomas and high-grade gliomas.

## Discussion

The present study showed that FLNC protein expression increased according to tumor histological grade in glioma and the circulating anti-FLNC autoantibody was induced in low-grade gliomas or in an early stage of glioma progression. In spite of the immunological privileged nature of the brain, the aberrant FLNC production could induce immune reaction. The induced serum autoantibody level decreased in high-grade gliomas or in a late stage of tumor progression. The patients who had induced strong immune reaction with high level of circulating anti-FLNC autoantibody survived longer than those with weak immune reaction. Antitumor immune responses against glioma antigens efficiently worked as immune-surveillance system in the early stage of gliomagenesis. These results collectively provide an evidence that serum anti-FLNC autoantibody can be a potential serum biomarker for early diagnosis of low-grade gliomas. However, a large-scale prospective study is required to confirm the usefulness of autoantibody- based biomarkers for gliomas.

Filamins are large cytoplasmic proteins whose dimers provide cells with mechanical resilience by cross-linking the actin filaments into dynamic three-dimensional structures [11-14]. Filamin A and B are ubiquitous, whereas FLNC is a muscle-restricted isoform. However, FLNC abundantly exists in both fetal brains and spinal cords [11,12]. Since many signaling molecules and receptors bind to the C-terminal region of filamins, it is now becoming clear that filamin is not merely a cytoskeletal support protein, but an important bridge between architecture and intracellular molecular signaling [23]. The C-terminal half of filamin A is reported to be a docking site for many intracellular signaling molecules, such as RalA, Rac, Rho, and Cdc42, and membrane receptors [24,25]. FLNC also binds some trans-membrane proteins such as sarcoglycan, presenilin, caveolin-1, and β_1_ integrin [26-29]. Recruitment of these signaling molecules to the C-terminal region of filamin facilitates their functional communication [23]. All these molecular hub function of filamins contributes to cancer progression, including metastasis and DNA damage response [15-22,30].

Actually, serum autoantibodies against filamins were reported to be detected in several cancers including colon and breast cancers [22]. In contrast, the central nervous system (CNS) is known to be an immunological privileged site and it was believed that antibody responses are only rarely elicited by the broad spectrum of antigens expressed by glioma [31]. We have shown that immune responses effectively operate even for intracerebral antigens when the tumor was in an early stage. The antibody response was gradually suppressed as tumor malignancy progressed. Not the inherent immune privilege of the CNS but the acquired immunosuppressive effect in glioma progression was the main cause of the suppressed FLNC antibody response in high-grade gliomas. However, the precise mechanism of the discrepancy between the higher tissue expression of FLNC in high-grade gliomas and the lower serum antibody level in these patients remained to be known. Local immunosuppression induced by T-cell specific lymphocytepenia is well documented in high-grade gliomas [31]. The impaired immunity is first occurred by immunosuppressive factors secreted by the tumors, such as vascular endothelial growth factor (VEGF), interleuikin-10 (IL-10), transforming growth factor-β (TGF-β), prostaglandin E_2_ (PGE_2_). Affected monocytes make a shift in cytokine secretion to favor a decrease in Th1-type cytokines and increase in Th2-type cytokines to induce T-cell signaling defect, IL-2 defect in T-cells, apoptosis of T-cell, and natural killer (NK) cell activity depression [31-33]. The long-term antigen exposure from a large tumor could induce immune tolerance through development of immune resistant tumor variants and the tumor microenvironment inducing immune cell anergy or death [34-36]. The induction and suppression of circulating anti-FLNC autoantibody would be individually regulated by the complex interaction between the host immune system and the tumor biology.

## Conclusion

In conclusion, we identified a novel glioma antigen, FLNC, utilizing SEREX and found that its autoantibody level is elevated in low-grade glioma patients compared with normal volunteers and high-grade glioma patients. Serum anti-FLNC autoantibody is thereby indicated to be a novel serum marker for the early diagnosis of low-grade gliomas. The conclusions of this study are limited by the small sample size and should be confirmed in a larger prospective study with an independent cohort of patients.

## Competing interests

The authors declare that they have no competing financial interests.

## Authors’ contribution

Designed the experiments: TH, MT, NS, YI. Performed the experiments: MH-A, AA, NS, TM. Analyzed the data: TM, TH, YI. All authors read and approved the final manuscript.

## Pre-publication history

The pre-publication history for this paper can be accessed here:

http://www.biomedcentral.com/1471-2407/14/452/prepub

## Supplementary Material

Additional file 1: Table S1 Candidate glioma antigens identified with SEREX. Click here for file
